# Cancer Risk in Diagnostic Radiation Workers in Korea from 1996–2002

**DOI:** 10.3390/ijerph10010314

**Published:** 2013-01-14

**Authors:** Kyung-Hwa Choi, Mina Ha, Won Jin Lee, Seung-Sik Hwang, Meeseon Jeong, Young-Woo Jin, Hyeog Ju Kim, Kwang-Yong Lee, Jung-Eun Lee, Jong-Won Kang, Heon Kim

**Affiliations:** 1 Department of Public Health, Graduate School of Dankook University, 119 Dandae-ro, Dongnam-gu, Cheonan, Chungnam 330-714, Korea; E-Mail: rosach72@hanmail.net; 2 Department of Preventive Medicine, Dankook University College of Medicine, 119 Dandae-ro, Dongnam-gu, Cheonan, Chungnam 330-714, Korea; 3 Department of Preventive Medicine, Korea University, 145 Anam-ro, Seongbuk-gu, Seoul 136-701, Korea; E-Mail: leewj@korea.ac.kr; 4 Department Preventive Medicine, Inha University, 100 Inha-ro, Nam-gu, Incheon 402-751, Korea; E-Mail: cyberdoc73@gmail.com; 5 Radiation Health Research Institute, Korea Hydro & Nuclear Power Co. Ltd., Wooichun-ro 308, Dobong-gu, Seoul 132-703, Korea; E-Mails: mjeong@khnp.co.kr (M.J.); ywjin@khnp.co.kr (Y.-W.J.); 6 Radiation Safety Division, Korea Food & Drug Administration, 187 Osongsaengmyeong2-ro, Cheongwon-gun, Chungbuk 363-700, Korea; E-Mails: khjtree@kfda.go.kr (H.J.K.); lky625@kfda.go.kr (K.-Y.L.); kdrlee@kfda.go.kr (J.-E.L.); 7 Department of Preventive Medicine, School of Medicine, Chungbuk National University, 52 Naesudong-ro, Heungdeok-gu, Cheongju, Chungbuk 361-763, Korea; E-Mail: jwkwdk@hanmail.net

**Keywords:** cancer risk, diagnostic radiation workers, effective dose

## Abstract

This study was aimed to examine the association between the effective radiation dose of diagnostic radiation workers in Korea and their risk for cancer. A total of 36,394 diagnostic radiation workers (159,189 person-years) were included in this study; the effective dose and cancer incidence were analyzed between the period 1996 and 2002. Median (range) follow-up time was 5.5 (0.04–7) years in males and 3.75 (0.04–7) years in females. Cancer risk related to the average annual effective dose and exposure to more than 5 mSv of annual radiation dose were calculated by the Cox proportional hazard model adjusted for occupation and age at the last follow-up. The standardized incidence ratio of cancer in radiation workers showed strong healthy worker effects in both male and female workers. The relative risk of all cancers from exposure of the average annual effective dose in the highest quartile (upper 75% or more of radiation dose) was 2.14 in male workers (95% CI: 1.48–3.10, *p*-trend: <0.0001) and 4.43 in female workers (95% CI: 2.17–9.04, *p*-trend: <0.0001), compared to those in the lower three quartiles of radiation exposure dose (less than upper 75% of radiation dose). Cancer risks of the brain (HR: 17.38, 95% CI: 1.05–287.8, *p*-trend: 0.04) and thyroid (HR: 3.88, 95% CI: 1.09–13.75, *p*-trend: 0.01) in female workers were significantly higher in the highest quartile group of radiation exposure compared to those in the lower three quartiles, and the risk of colon and rectum cancers in male workers showed a significantly increasing trend according to the increase of the average annual radiation dose (HR: 2.37, 95% CI: 0.99–5.67, *p*-trend: 0.02). The relative risk of leukemia in male workers and that of brain cancer in female workers were significantly higher in the group of people who had been exposed to more than 5 mSv/year than those exposed to less than 5 mSv/year (HR: 11.75, 95% CI: 1.08–128.20; HR: 63.11, 95% CI: 3.70–1,075.00, respectively). Although the present study involved a relatively young population and a short follow-up time, statistically significant increased risks of some cancers in radiation workers were found, which warrants a longer follow-up study and more intensive protective measures in this population.

## 1. Introduction

Radiation is increasingly used in modern medicine since the introduction of numerous new radiologic procedures [1]. There are about 2.3 million diagnostic radiation workers worldwide and the number has been rapidly increasing [1,2,3]. Radiation exposure of radiological technologists is about two times higher than that of other occupation groups in the fields of diagnostic radiation workers, such as physicians, dentists, dental hygienists, and nurses [3,4].

In Korea, 15,197 workers in 1996 and 41,108 in 2006 working in the diagnostic radiation fields were monitored and reported regarding their occupational radiation exposure [3]. Although the average annual effective radiation dose of the monitored diagnostic radiation workers in Korea from 1996 to 2006 showed a decreasing tendency [3], the levels were about double the reported worldwide average in the early 1990s [4]. Diagnostic radiation workers are typically exposed to low doses at low dose rates to most areas of the body, which allows the assessment of cancer risk for many organs and tissues. However, health effects associated with occupational radiation exposure in Korean diagnostic radiation workers have not yet been assessed. Therefore, this study was aimed to examine the association between the monitored effective radiation dose of diagnostic radiation workers and their cancer risk.

## 2. Materials and Methods

### 2.1. Study Subjects

The study subjects included diagnostic radiation workers who were being monitored for ionizing radiation exposure at workplace and registered in the national dose registry of the Korean Food and Drug Administration (KFDA) maintains [5]. Information in the dose registry includes name, gender, date of birth, personal identification number (PID), job classification, quarterly recorded radiation dose, and the beginning and end of the measurement period. A total of 65,353 persons were recorded in 2006.

**Figure 1:**
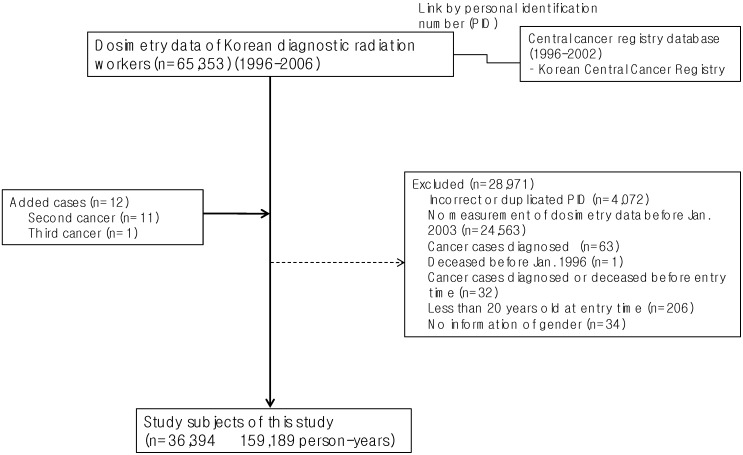
Selection process of the study subjects, Korea, 1996–2002.

A total of 36,394 diagnostic radiation workers (159,189 person-years) were analyzed in this study after excluding those with incorrect or duplicated PID (4,072 persons), those who were not employed by Jan 2003 or whose dosimetry data were missing before Jan 2003 (24,563 persons), previous cancer diagnosis (see “identification of cancer occurrence” section) before January 1996 (63 persons), deceased before January 1996 (one person), cancer diagnosis before entry time (32 persons), aged less than 20 years (206 persons), and missing information on gender (34 persons) (Figure 1). Of 36,394 workers, 13,076 were radiologic technologists (35.9%), 7,658 dentists (21.0%), 3,324 physicians (9.1%), 1,806 dental hygienists (5.0%), and 10,530 other types of diagnostic radiation workers (28.9%; including nurses, assistant nurses, and others).

### 2.2. Identification of Cancer Occurrence

We identified persons who were diagnosed with cancer from 1 January 1996 to 31 December 2002 among the recorded persons in the dose registry by linkage with the Korea Central Cancer Registry (KCCR) using the PID. The Mortality/Incidence ratio was 54.5% and Death Certificate Only was 4.6% in KCCR in 2002 [6]. A total of 192 cancers occurred and 260 died during the follow-up period.

### 2.3. Radiation Dose

We used the national dose registry of the KFDA for diagnostic radiation workers. The personal radiation exposure dose of each diagnostic radiation worker was quarterly measured using a thermoluminescence dosimeter (TLD) [3]. All instruments at four personal monitoring centers were calibrated annually. A survey performed in 2008 showed that 76.3% of radiologic technologists to whom TLD dosimeters were issued reported “always wear TLD at workplace from 1990 to 2008” [7].

The deep radiation dose was calculated using the surface recorded dose by the methods presented in the Enforcement Regulations of Nuclear Safety Act of the National Committee of Nuclear Safety [8]. We calculated the annual average and maximum effective dose during the period from 1996 to 2002 for each individual in the total of 36,394 study subjects.

### 2.4. Potential Confounding or Modifying Factors

As potential confounding or modifying factors, we considered attained age (age at the end of follow-up), gender and job classification (radiologic technologists, physicians, dentists, dental hygienists, and others) to estimate the association between occupational radiation exposure and cancer occurrence. Gender was taken into account as a variable for stratification; attained age and job classification were included in all adjusted models for the estimation of cancer risk as covariates.

### 2.5. Statistical Analysis

To examine the cancer risk of diagnostic radiation workers in Korea, we used two comparison groups, *i.e.*, external and internal comparison groups. As the external comparison group, we used the Korean general population and their cancer incidence. The standardized incidence rates (SIRs) and 95% confidence intervals [9] of all cancers and radiation-induced cancers [10] were calculated according to specific gender and age by calendar year from 1999 to 2002 due to the availability of nationwide rates from 1999 in Korea [11].

The second comparison group comprised of radiation workers themselves, *i.e.*, internal comparison, which allowed us to avoid the “healthy worker effect”, a commonly occurred selection bias in occupational epidemiology [12]. We compared cancer incidence in workers with a higher level and a lower level of radiation exposure. With reference of cancer incidence in workers with a relatively lower level of radiation exposure (0~75%), we estimated the cancer risk of workers with a higher level of radiation exposure (75%~100%). In addition, a continuous scale of exposure dose was used to estimate the cancer risk for unit increase of radiation dose. We also estimated the cancer risk of workers who ever had experienced exposure to radiation more than 5mSv per year, compared to those never experienced. And we estimated the cancer risk for the frequency of experiences of exposure with more than 5mSv per year*.* The age at entry was defined as the age at the first recorded month. In cases of workers who started their work in 1996 or before 1996, the age at entry was defined as their age in 1996. The year of follow-up for each person was defined as the duration from entry to the time of cancer identification by KCCR, 31 December 2002, or time of death without cancer diagnosis according to the Death Certificate [11].

The cancer risk for the effective dose was calculated as the hazard risk and 95% confidence intervals using the Cox proportional hazard model adjusted for occupation and age at the end of follow-up. All analyses were conducted using R [13] with a significance level of 0.05.

## 3. Results

### 3.1. Distribution of Radiation Effective Dose (1996–2002)

The mean (SD) age at entry was 34.11 (9.36) in males and 27.16 (5.85) in females, and was significantly different according to occupation. The median (range) follow-up duration was 5.5 (0.04–7) years in males and 3.8 (0.04–7) years in females. Five% of male workers and 1% of female workers experienced more than 5 mSv/year of radiation exposure. Radiological technologists showed the highest proportion of having been exposed to more than 5 mSv/year: 8.5 and 1.4% in male and female workers, respectively. The radiation effective dose in radiologic technologists was the highest among diagnostic workers in the annual average and annual maximum. The radiation effective dose in male workers was higher than that of female workers (Table 1).

### 3.2. Standardized Incidence Rates (SIRs, 1999–2002)

Standardized incidence rates (SIRs) for all cancers, stomach, colon and rectum, liver, lung, bladder, non-Hodgkin’s lymphoma, all cancers excluding leukemia, total radiation related cancers, and total radiation related cancers excluding leukemia were significantly less than 1 in diagnostic radiation workers, when compared to the incidence of cancers in the age-adjusted general population of males in Korea. In female workers, the incidence of all cancers, stomach, all cancers excluding leukemia total radiation related cancers, and total radiation cancers excluding leukemia were significantly lower in diagnostic radiation workers than those in the age-adjusted general population of females in Korea (Table 2).

### 3.3. Average Annual Effective Dose and Cancer Risk

The risk of all cancers, all cancers excluding leukemia, total radiation related cancers, and total radiation related cancers excluding leukemia from exposure of the average annual effective dose of radiation in the highest quartile dose group was significantly higher than that in the lower three quartiles dose group in a significant dose-response manner. In male workers, the risk of colon and rectum cancers showed a significant increasing trend according to the increasing average annual effective dose. In female workers, the risk of brain and thyroid cancers in the highest quartile dose group was significantly higher than that in the lower three quartiles dose group in a significant dose-response manner (Table 3). When we repeatedly analyzed the data using a continuous scale of average annual effective dose, the pattern of the results was not materially changed, but the statistical significances were weakened as shown in Table 3. Repeated analyses for the maximum annual dose did not show any significant results (Data not shown).

**Table 1:** Occupational radiation exposure doses in diagnostic radiation workers by profession, Korea, 1996–2002.

		N	Follow-up time (person-years)	Age at entry (years)	Average annual effective dose			Maximum annual effective dose			> 5 mSv/year	
				Mean (SD)	Q1	Median	Q3	Q1	Median	Q3	N	%
**Male workers**												
All		25,022	114,339	34.11 (9.36)	0.14	0.41	1.26	0.26	0.83	2.90	1,249	5.0
	Radiologic technologists	9,741	49,395	29.89 (6.75)	0.52	1.17	2.70	1.04	2.49	5.61	828	8.5
	Physicians	2,700	9,865	36.80 (6.87)	0.06	0.13	0.25	0.08	0.22	0.48	9	0.3
	Dentists	6,782	26,558	39.62 (8.78)	0.10	0.23	0.59	0.14	0.38	1.17	147	2.2
	Dental hygienists	9	30	31.11 (7.51)	0.04	0.09	0.17	0.08	0.12	0.22	0	0.0
	Others	5,790	28,491	33.50 (10.99)	0.08	0.24	0.56	0.22	0.57	1.66	265	4.6
**Female workers**												
All		11,372	44,850	27.16 (5.85)	0.07	0.21	0.52	0.13	0.39	1.12	118	1.0
	Radiologic technologists	3,335	12,893	25.40 (4.87)	0.21	0.47	0.98	0.33	0.77	2.00	48	1.4
	Physicians	624	2,114	32.27 (5.40)	0.05	0.12	0.23	0.06	0.19	0.42	1	0.2
	Dentists	876	3,411	33.32 (6.19)	0.12	0.30	0.68	0.17	0.50	1.62	9	1.0
	Dental hygienists	1,797	3,800	25.23 (3.76)	0.04	0.12	0.25	0.05	0.14	0.35	0	0.0
	Others	4,740	22,632	27.31 (5.91)	0.05	0.15	0.37	0.14	0.37	0.96	60	1.3

Q1, Q3: the values at the lower 25 and 75 percentiles of the radiation dose distribution.

**Table 2:** Standardized incidence ratio (SIR) by gender in diagnostic radiation workers, Korea, 1999–2002.

Cancer		Male (n = 24,942; 114,216 person-year)				Female (n = 11,360; 44,829 person-year)			
		No. of cancer		SIR ^a^	95% CI	No. of cancer		SIR ^a^	95% CI
		Observed	Expected			Observed	Expected		
All (C00-C97)		110	297.13	0.37	0.30 to 0.44	32	57.05	0.56	0.37 to 0.76
	Esophagus (C15)	4	6.42	0.62	0.01 to 1.23	0	0.04	-	-
	Stomach (C16)	21	73.60	0.29	0.16 to 0.41	1	6.94	0.14	−0.14 to 0.43
	Colon and rectum (C18-C20)	16	30.65	0.52	0.27 to 0.78	2	2.94	0.68	−0.26 to 1.62
	Liver (C22)	15	58.25	0.26	0.13 to 0.39	1	1.56	0.64	−0.61 to 1.90
	Lung (C33-C34)	7	38.22	0.18	0.05 to 0.32	0	1.33	-	-
	Breast (C50)	0	0.31	-	-	10	12.63	0.79	0.30 to 1.28
	Bladder (C67)	3	7.88	0.38	−0.05 to 0.81	0	0.18	-	-
	Brain (C70-C72)	1	4.58	0.22	−0.21 to 0.65	2	1.04	1.92	−0.74 to 4.59
	Thyroid (C73)	7	4.82	1.45	0.38 to 2.53	9	9.27	0.97	0.34 to 1.61
	Hodgkin (C81)	1	0.63	1.58	−1.51 to 4.67	0	0.17	-	-
	Non-Hodgkin lymphoma (C82-C85, C96)	2	8.35	0.24	−0.09 to 0.57	0	1.24	-	-
	Leukemia (C91-C95)	4	6.91	0.58	0.01 to 1.15	1	1.73	0.58	−0.56 to 1.71
**All cancer** **s excluding leukemia**		106	290.22	0.37	0.30 to 0.43	31	55.32	0.56	0.36 to 0.76
**Total radiation related cancer** **s ^b^**		81	240.63	0.34	0.26 to 0.41	26	39.07	0.67	0.41 to 0.92
**Total radiation related cancer** **s excluding leukemia**		77	233.72	0.33	0.26 to 0.40	25	37.34	0.67	0.41 to 0.93

^a^ Standardized incidence ratios and 95% confidence intervals were indirectly standardized by age and year of diagnosis of the Korean general population (Korean Statistical information service, 1999–2002) using Armitage and Berry methods. ^b^ Total radiation related cancers included cancers in esophagus (C15), stomach (C16), colon and rectum (C18-C20), liver (C22), lung (C33-C34), breast (C50), bladder (C67), brain (C70-C72), thyroid (C73), Hodgkin’s disease (C81), non-Hodgkin’s lymphoma (C82-C85, C96), and leukemia (C91-C95).

**Table 3:** Hazard ratio and 95% confidence intervals of cancer for average annual effective dose in diagnostic radiation workers, Korea, 1996–2002.

Cancer		Average of annual effective dose											
		Male (n = 25,022 114,339 person-years)						Female (n = 11,372 44,850 person-years)					
		Q1-Q3 (n = 17,412)	Q4 (n = 7,610)			*p*-trend ^a^	*p*-trend ^b^	Q1-Q3 (n = 9,883)	Q4 (n = 1,489)			*p*-trend ^a^	*p*-trend ^b^
		Case	Case	HR	95% CI			Case	Case	HR	95% CI		
**All (C00-C97)**		104	50	2.14	1.48–3.10	<0.0001	0.01	25	13	4.43	2.17–9.04	<0.0001	0.06
	Esophagus (C15)	5	1	1.78	0.21–15.27	0.68	0.84	0	0	-	-	-	
	Stomach (C16)	20	7	0.97	0.38–2.49	0.65	0.57	2	1	5.99	0.53–68.19	0.22	0.94
	Colon and rectum (C18-C20)	19	8	2.37	0.99–5.67	0.02	0.82	1	1	10.25	0.54–192.77	0.09	0.32
	Liver (C22)	17	6	1.99	0.73–5.42	0.10	0.83	1	0	0.00	0–Inf	<0.0001	0.96
	Lung (C33-C34)	7	3	2.89	0.70–11.96	0.14	0.26	0	0	-	-	-	
	Breast (C50)	-	-	-	-	-		7	3	3.34	0.78–14.32	0.06	0.59
	Bladder (C67)	3	1	0.69	0.07–6.63	0.99	0.61	0	0	-	-		
	Brain (C70-C72)	1	1	5.59	0.35–90.42	0.19	0.08	1	1	17.38	1.05–287.80	0.04	0.40
	Thyroid (C73)	4	5	3.55	0.85–14.81	0.05	0.12	8	4	3.88	1.09–13.75	0.01	0.13
	Hodgkin (C81)	1	1	5.00	0–Inf	0.21	0.91	0	0	-	-	-	
	Non-Hodgkin (C82-C85, C96)	2	1	0.63	0–Inf	0.87	0.36	1	0	0.00	0–Inf	-	0.93
	Leukemia (C91-C95)	3	1	3.48	0.36–34	0.32	0.80	1	0	0.00	0–Inf	0.48	0.99
**All cancers excluding leukemia**		101	49	2.11	1.45–3.08	<0.0001	0.01	24	13	4.53	2.21–9.29	<0.0001	0.06
**Total radiation related cancers ^c^**		82	35	1.95	1.27–3.00	<0.0001	0.01	22	10	4.29	1.92–9.58	<0.0001	0.06
**Total radiation related cancers excluding leukemia**		79	34	1.91	1.23–2.96	0.0002	0.01	21	10	4.41	1.96–9.91	<0.0001	0.06

Q1-Q3; 0–0.952 mSv, Q4; 0.952–569 mSv. Hazard Ratios and 95% confidence intervals for Q4 group calculated using the Cox proportional hazard model adjusted for age at the last follow-up time and occupation referenced by Q1-Q3 group. ^a ^*p*-trend for radiation dose category (Q1, Q2 and Q3, and Q4) as an ordinal scale calculated using the corresponding model. ^b ^*p*-trend for continuous scale of radiation dose calculated using the corresponding model. ^c^ Total radiation related cancers include cancers in the esophagus (C15), stomach (C16), colon and rectum (C18-C20), liver (C22), lung (C33-C34), breast (C50), bladder (C67), brain (C70-C72), thyroid (C73), Hodgkin’s disease (C81), non-Hodgkin’s lymphoma (C82-C85, C96), and leukemia (C91-C95).

**Table 4:** Hazard ratio and 95% confidence intervals of cancer for experience exposed to more than 5 mSv of annual radiation dose in diagnostic radiation workers, Korea, 1996–2002.

Cancer		Experience of annual doses exceeding 5 mSv											
		Male (n = 25,022 114,339 person-years)						Female (n = 11,372 44,850 person-years)					
		No (n = 23,773)	Yes (n = 1,249)			Frequency of experience ^c^		No (n = 11,254)	Yes (n = 118)			Frequency of experience ^c^	
		Case	Case	HR ^a^	95% CI	HR ^b^	95% CI	Case	Case	HR ^a^	95% CI	HR ^b^	95% CI
**All (C00-C97)**		147	7	0.80	0.37–1.72	0.79	0.43–1.45	37	1	1.20	0.16–9.05	1.05	0.17–6.39
	Esophagus (C15)	6	0			0.00	0–Inf	0	0	-	-	-	-
	Stomach (C16)	25	2	1.07	0.25–4.60	0.88	0.26–2.99	3	0				
	Colon and rectum (C18-C20)	26	1	0.83	0.11–6.16	0.73	0.13–4.07	2	0				
	Liver (C22)	22	1	0.90	0.12–6.78	0.77	0.14–4.23	1	0	-		1.04	0–Inf
	Lung (C33-C34)	10	0					0	0	-	-		
	Breast (C50)	0	0					10	0				
	Bladder (C67)	4	0					0	0			-	-
	Brain (C70-C72)	2	0					1	1	63.11	3.70–1075	13.2	1.80–96.76
	Thyroid (C73)	9	0					12	0				
	Hodgkin (C81)	2	0					0	0				
	Non-Hodgkin (C82-C85, C96)	3	0					1	0			1.02	0–Inf
	Leukemia (C91-C95)	3	1	11.75	1.08–128.2	4.67	0.30–71.66	1	0			0.90	0–Inf
**All cancers excluding leukemia**		144	6	0.69	0.30–1.57	0.72	0.37–1.39	36	1	1.21	0.16–9.09	1.05	0.17–6.37
**Total radiation related cancers ^d^**		112	5	0.77	0.31–1.90	0.69	0.32–1.50	31	1	1.91	0.26–14.15	1.47	0.26–8.25
**Total radiation related cancers excluding leukemia**		109	4	0.62	0.23–1.70	0.59	0.25–1.41	30	1	1.92	0.26–14.26	1.47	0.26–8.21

^a^ Hazard Ratios and 95% confidence intervals for the group ever experienced more than 5 mSv/year radiation exposure calculated using the Cox proportional hazard model adjusted for age at the last follow-up time and occupation referenced by the group never experienced more than 5 mSv/year radiation exposure. ^b^ Hazard Ratios and 95% confidence intervals for the frequency of experience with more than 5 mSv of radiation exposure as a continuous scale in the corresponding model. ^c^ The frequency of experience with more than 5 mSv of radiation exposure during the follow-up period. ^d^ Total radiation related cancers include cancers in the esophagus (C15), stomach (C16), colon and rectum (C18-C20), liver (C22), lung (C33-C34), breast (C50), bladder (C67), brain (C70-C72), thyroid (C73), Hodgkin’s disease (C81), non-Hodgkin’s lymphoma (C82-C85, C96), and leukemia (C91-C95).

### 3.4. Experience Exposed to more than 5 mSv/Year and Cancer Risk

The total number of subjects exceeding 5mSv a year was 1,249 male workers and 118 female workers (Table 4).Among them, 281 male (22.5%) and 14 female (11.9%) workers had experienced two times of exposure during the follow-up period while the remaining had just one exposure. The mean dose when exceeding 5 mSv a year was 17.43 (median 8.39) and 8.57 mSv (median 2.55) in 1,249 male and 118 female workers, respectively. The highest annual exposure dose was 569 mSv in this study. The worker with the highest exposure dose was a male radiological technologist in his 20’s and had been working in the emergency department of a hospital as military service at the time when his monitored doses were registered.

None of the specific cancer types or groups of cancers showed increased risk in workers with more than 5 mSv of annual dose exposure compared to those less than 5 mSv of annual expose except for leukemia in males and brain tumor in females. The risk of leukemia was significantly increased in male workers with more than 5 mSv of annual exposure compared to those without exposure although the number of leukemia cases was only 4. The diagnosis of the 4 leukemia patients was C920 (Acute myeloid leukemia, 2 persons) and C921 (Chronic myeloid leukemia, 2 persons) in males. The risk of brain cancer was significantly increased in female workers with more than 5 mSv of annual exposure compared to those without exposure although the number of brain tumor cases was only 2. The ICD code of the 2 brain cancer patients was C711 (Frontal lobe) and C718 (Overlapping lesion of brain). The results of the analyses using the frequency of exposure of the annual dose exceeding 5mSv as a continuous exposure variable showed similar findings with an improvement of statistical stability (Table 4).

## 4. Discussion

We found that the risk of all cancers in radiation workers, particularly leukemia in male workers, and cancers of the brain and thyroid in female workers with exposure from the higher radiation dose were significantly increased compared to those with exposure of the lower radiation dose in the present study involving relatively young subjects with a short follow-up period.

Leukemia can be an early cancer occurring 2–5 years after exposure to radiation [14]. In the male workers of the present study, the median follow-up duration was 5.5 years with the relatively sufficient power of person-years. Therefore, a significantly increased risk of leukemia was found while other solid cancers were not detected. In a U.S. radiologic technologist cohort study, the relative risk of acute leukemia for the group of people who experienced more than 50 times of radiography was 2.6 (1.3–5.4) times higher than those who experienced less than 50 times [2]. Although a direct comparison is not appropriate due to the lack of dosimetry in the USRT Health study, the results are compatible with the present study. 

Brain tumor is one of the cancers possibly induced by radiation exposure [2,10,15,16,17]. Although only two cases of brain tumor were found in the present study, the risk in female workers with more than 5 mSv of annual exposure was significantly increased compared to those with no experience. Brain tumor is a rare type of cancer among solid cancers which makes it difficult to obtain sufficient statistical power in medium-sized epidemiological studies. The risk of brain tumor showed a consistently increasing trend according to occupational radiation exposure in a previous hospital worker study [17]. The risk of brain tumor was also slightly increased in the Life Span Study (LSS) cohort of Atomic Bomb Survivors [16].

Thyroid cancer is one of the well known cancers related to ionizing radiation exposure and for more frequent occurrence in women [16]. In the LSS cohort, the excess relative risk for thyroid cancer was 0.49/Gy (90% CI: 0.15–1.15) in males, and 0.65/Gy (0.27–1.25) in females [10]. An increased risk of thyroid cancer in medical workers in China [18] and USRT [15] were reported with suggestion of a possible effect of earlier detection among medical workers with easy access to health care [15]. In Korean nuclear workers, the increasing trend of thyroid cancer incidence according to the increase of cumulative dose also indicated a possible over-diagnosis due to periodic health examinations (*p*-trend = 0.0307) [19]. The findings of thyroid cancer risk in the present study that showed a significantly increased risk in female workers and an increasing trend in male workers may reflect, at least in part, a possible over-diagnosis as well.

An interesting finding is the fact that the occupational exposure level in the study subjects is very low (median annual exposure average: 0.38 mSv in the present study). Considering the fact that previous studies reported a significantly increased cancer risk among early workers who might have been exposed to high levels of ionizing radiation in the workplace [15], the positive findings in the present study among workers exposed to a very low dose of radiation suggest an important public health implication.

The so-called healthy worker effect is a commonly found phenomenon in occupational epidemiology [20], which is reflected in the standardized rate ratio as being less than one when compared to the external control, *i.e.*, the general population. In a Canadian diagnostic radiation worker study, SIR for all cancers except nonmelanoma skin cancers was 0.86 in males and 0.64 in females [1]. The SIRs of all cancers in diagnostic workers in Korea were lower compared to those values from the Canadian study (0.37 in males, 0.56 in females), showing a stronger healthy worker effect. On the other hand, the USRT study showed a higher SIR in radiological technologists with the SIR of all cancers in females (1.07, 1.03–1.11), all solid cancers (1.06, 1.02–1.10), breast cancer (1.16, 1.09–1.23), melanoma (1.66, 1.43–1.89), and thyroid cancer (1.54, 1.24–1.83), which may be mainly attributed to the high dose radiation exposure in early workers particularly before the 1950s [15]. On the other hand, the calculated SIRs using an internal comparison, in which the healthy worker effect may not play a major role, showed a significantly increased risk of cancer in radiation workers. In a Chinese X-ray male worker study, the SIR of all cancers was 1.24, and for leukemia was 2.29, showing statistical significance compared to non-radiology medical workers [1].

This study has some limitations. First, cancer risks were estimated after adjustment for confounding or modifying factors such as gender, age, and occupation, but alcohol intake, smoking and other factors such as socioeconomic positions were not considered. However, such potential confounders, *i.e.*, health behaviors, are known to be typically highly correlated with the type of occupation in the occupationally exposed radiation worker population which would then be correlated with radiation exposure dose [21]. Second, the minimum latent period for most solid cancers is 10–20 years [21]. The follow-up period of 3.7 to 5.5 years in the present study may not be sufficient to estimate the risk of solid cancers except for leukemia which is a type of early occurring cancer. There might be no need to consider the lag time in the data analysis of the present study because we used the average annual dose instead of cumulative dose. Further follow-up studies with longer follow-up periods with consideration of lag time of cancer occurrence in relation to the cumulative exposure dose are warranted. Third, in the radiation exposure dose, we did not take into account the behavior pattern of wearing the TLD dosimeter. About less than 80% of radiologic technologists in Korea are reported to “always” wear the dosimeter at work [7], suggesting that the radiation dose recorded in the dosimetry registry is possibly underestimated in this population. Fourth, we could not use the data of radiation effective dose before 1996 due to the lack of data availability. Presumably, the exposure dose before 1996 might be higher than that after 1996 because 1996 was the year when the law of radiation protection for radiation workers was enforced and also the year when systemic protection activities by the government including exposure monitoring for all radiation workers in hospitals using the TLD dosimeter were started [5]. Therefore, there might be a possible misclassification of cumulative exposure dose, particularly in aged workers who started radiation work at an earlier time. In this study, instead of using the cumulative exposure dose for total working periods since 1996, the dosimetry data became available, we used the annual average effective dose and exposure level of 5mSv or more per year in estimating the cancer risk to avoid misclassification of the cumulative exposure dose. Furthermore, we do not have information of the years before 1996, and we could not use the total duration of being employed as an exposure surrogate as well. Fifth, although we estimated cancer risks for various organs, information on organ specific dose was not available in the present study. Sixth, radiation exposure from other sources is one of the important confounding factors in the estimation of cancer risk due to occupational radiation exposure. Information on medical radiation exposure from health examination, disease diagnosis or radiotherapy, and/or natural radiation exposure from residing in radon-rich regions, *etc.*, was not available and could not be taken into account in the present study. However, this would not likely affect our results differently. Last, it was difficult to obtain an appropriate external control group to compare the cancer occurrence because of a strong healthy worker effect in this study population as mentioned above. Using an internal comparison method, *i.e.*, comparison between subgroups within the study population according to the level of radiation exposure dose, we estimated the relative risk of cancer occurrence.

Despite the limitations mentioned above, this is the first study to evaluate cancer risk in relation to the quarterly measured radiation dose in Korean diagnostic radiation workers. The results warrant future research involving a longer follow-up period with information on potential confounding factors.
